# Two distinct cellular pathways leading to endothelial cell cytotoxicity by silica nanoparticle size

**DOI:** 10.1186/s12951-019-0456-4

**Published:** 2019-02-05

**Authors:** Kyungmin Lee, Jangwook Lee, Minjeong Kwak, Young-Lai Cho, Byungtae Hwang, Min Ji Cho, Na Geum Lee, Jongjin Park, Sang-Hyun Lee, Jong-Gil Park, Yeon-Gu Kim, Jang-Seong Kim, Tae-Su Han, Hyun-Soo Cho, Young-Jun Park, Seon-Jin Lee, Hee Gu Lee, Won Kon Kim, In Cheul Jeung, Nam Woong Song, Kwang-Hee Bae, Jeong-Ki Min

**Affiliations:** 1 Biotherapeutics Translational Research Center, Korea Research Institute of Bioscience and Biotechnology (KRIBB), 125 Gwahak-ro, Yuseong-gu, Daejeon, 34141 Republic of Korea; 2 Center for Nano-Bio Measurement, Korea Research Institute of Standards and Science (KRISS), 267 Gajeong-ro, Yuseong-gu, Daejeon, 34113 Republic of Korea; 3 Research Center for Metabolic Regulation, KRIBB, 125 Gwahak-ro, Yuseong-gu, Daejeon, 34141 Republic of Korea; 4 Department of Biomolecular Science, KRIBB School of Bioscience, Korea University of Science and Technology (UST), 217 Gajeong-ro, Yuseong-gu, Daejeon, 34113 Republic of Korea; 5 Stem Cell Research Center, KRIBB, 125 Gwahak-ro, Yuseong-gu, Daejeon, 34141 Republic of Korea; 6 Immunotherapy Convergence Research Center, KRIBB, 125 Gwahak-ro, Yuseong-gu, Daejeon, 34141 Republic of Korea; 7 Department of Obstetrics and Gynecology, College of Medicine, The Catholic University of Korea, 222 Banpo-daero Seocho-gu, Seoul, 06591 Republic of Korea; 8 Korea Research Institute of Standards and Science (KRISS), 267 Gajeong-ro, Yuseong-gu, Daejeon, 34113 Republic of Korea

**Keywords:** Silica nanoparticles, Apoptosis, Necroptosis, ROS, Autophagy

## Abstract

**Background:**

Silica nanoparticles (SiNPs) are widely used for biosensing and diagnostics, and for the targeted delivery of therapeutic agents. Safety concerns about the biomedical and clinical applications of SiNPs have been raised, necessitating analysis of the effects of their intrinsic properties, such as sizes, shapes, and surface physicochemical characteristics, on human health to minimize risk in biomedical applications. In particular, SiNP size-associated toxicological effects, and the underlying molecular mechanisms in the vascular endothelium remain unclear. This study aimed to elucidate the detailed mechanisms underlying the cellular response to exposure to trace amounts of SiNPs and to determine applicable size criteria for biomedical application.

**Methods:**

To clarify whether these SiNP-mediated cytotoxicity due to induction of apoptosis or necrosis, human ECs were treated with SiNPs of four different non-overlapping sizes under low serum-containing condition, stained with annexin V and propidium iodide (PI), and subjected to flow cytometric analysis (FACS). Two types of cell death mechanisms were assessed in terms of production of reactive oxygen species (ROS), endoplasmic reticulum (ER) stress induction, and autophagy activity.

**Results:**

Spherical SiNPs had a diameter of 21.8 nm; this was further increased to 31.4, 42.9, and 56.7 nm. Hence, we investigated these effects in human endothelial cells (ECs) treated with these nanoparticles under overlap- or agglomerate-free conditions. The 20-nm SiNPs, but not SiNPs of other sizes, significantly induced apoptosis and necrosis. Surprisingly, the two types of cell death occurred independently and through different mechanisms. Apoptotic cell death resulted from ROS-mediated ER stress. Furthermore, autophagy-mediated necrotic cell death was induced through the PI3K/AKT/eNOS signaling axis. Together, the present results indicate that SiNPs within a diameter of < 20-nm pose greater risks to cells in terms of cytotoxic effects.

**Conclusion:**

These data provide novel insights into the size-dependence of the cytotoxic effects of silica nanoparticles and the underlying molecular mechanisms. The findings are expected to inform the applicable size range of SiNPs to ensure their safety in biomedical and clinical applications.

**Electronic supplementary material:**

The online version of this article (10.1186/s12951-019-0456-4) contains supplementary material, which is available to authorized users.

## Background

Nanotechnology has enabled rapid progress in the fields of pharmacology and medicine. Numerous types of nanoparticles have been developed using various organic, inorganic, and hybrid materials [1]. Among these, silica is an attractive base inorganic material for engineered nanoparticles [2]. Silica nanoparticles (SiNPs) are generally of two types: rigid (nonporous) and mesoporous nanostructures. Rigid SiNPs have attracted increasing attention as an efficient host material for cellular cargo, typically enzymes, and they are usually immobilized via adsorption or covalent cross-linking methods [3]. Mesoporous silica nanoparticles have numerous pores that are suitable to load cargo. In addition, lipid bilayer coatings or organic modifications are applied at nanoparticle surfaces protection or release control of such cargo [4, 5]. Recently, various hybrid nanocomposites containing SiNPs have been synthesized and applied for controlled drug delivery and targeted imaging agents [6, 7]. Nonetheless, the potential risks of SiNPs on human heath have not yet been fully assessed.

Numerous studies on SiNP-related cytotoxicity have been conducted in various cell types including HaCat cells [8], myocardial cells [9], human embryonic kidney cells [10], HepG2 cells [11], macrophages [12], lung cancer cells [13], and endothelial cells (ECs) [14–16]. These reports have broadly addressed the risks and potential utility in biomedical applications based on the intrinsic factors of SiNPs such as their size, shape, and surface modifications. Notwithstanding conflicting data regarding their potential harmful effects on cells, these studies provide an in-depth insight into the size-dependent biological response of SiNPs. The majority of the results reported were obtained for SiNPs greater than 50 nm, in the presence of serum in which SiNPs are agglomerated [17]. Therefore, the effect of agglomeration-free conditions on SiNPs is yet unclear.

It should be noted that intravenously injected SiNPs first interact with the inner linings of the lumen blood vessels, which may affect vascular homeostasis and maintenance of function. Therefore, safety issues concerning potential risks to the ECs, during the systemic translocation of the SiNPs, should be investigated as priority. The induction of reactive oxygen species (ROS), inflammation, von Willebrand factor (VWF), lysosome activity, necrotic cell death, and autophagy has been reported in human primary blood components and ECs exposed to SiNPs [14, 18–20]. However, the biological response to and toxic effects of SiNPs remain poorly understood. Previous studies attempted to elucidate the interactions between SiNPs and ECs have focused on time- and dose-dependent biological effects rather than on the size-dependent effects. Furthermore, the detailed mechanisms underlying the size-dependent cytotoxicity of SiNPs in ECs are still unclear.

The endoplasmic reticulum (ER) is an important intracellular organelle involved in the secretory pathway. The ER regulates the biosynthesis of proteins or lipids and maintains calcium homeostasis. ER stress is induced by various pathophysiological conditions such as oxidative stress, glucose deprivation, DNA damage, and viral infection [21]. Toxicity mechanisms associated with oxidative stress, which induce apoptosis in SiNPs have recently been reported in various cell lines, demonstrating a crucial role for ROS. ROS-mediated oxidative stress is a common cytotoxic effect of non-degradable nanoparticles. However, there are few studies of the size-dependent toxicity on ECs of SiNPs less than 50 nm in size.

Autophagy, which is a catabolic processes involving survival/death, pathogen clearance, and antigen presentation [22], is considered an important lysosome-based pathway of necrosis. Autophagosomes markedly accumulate in various cell lines treated with nanoparticles. However, only a few studies have focused on the biological effect of SiNPs of 50 nm or less, in terms of the induction of autophagy. Moreover, the underlying roles of autophagy in SiNP-mediated cytotoxicity remain unclear.

The present study aimed to identify applicable size criteria for SiNPs and to elucidate the detailed mechanisms underlying the cellular response to exposure to trace amounts of SiNPs, to ensure safety in biological applications. We prepared four different SiNPs without overlap in size (20, 30, 40, and 50 nm) and treated them with ECs. The SiNPs of 20-nm showed cytotoxic effect even at a low concentration and decreased cell viability. The SiNPs of 20-nm in size were found to induce ROS-mediated ER stress and autophagy, leading to two types of programmed cell death, apoptosis and necroptosis, respectively. Surprisingly, the two types of cell death were found to occur independently and via different mechanisms.

## Results

### Characteristics of SiNPs

**Fig. 1:**
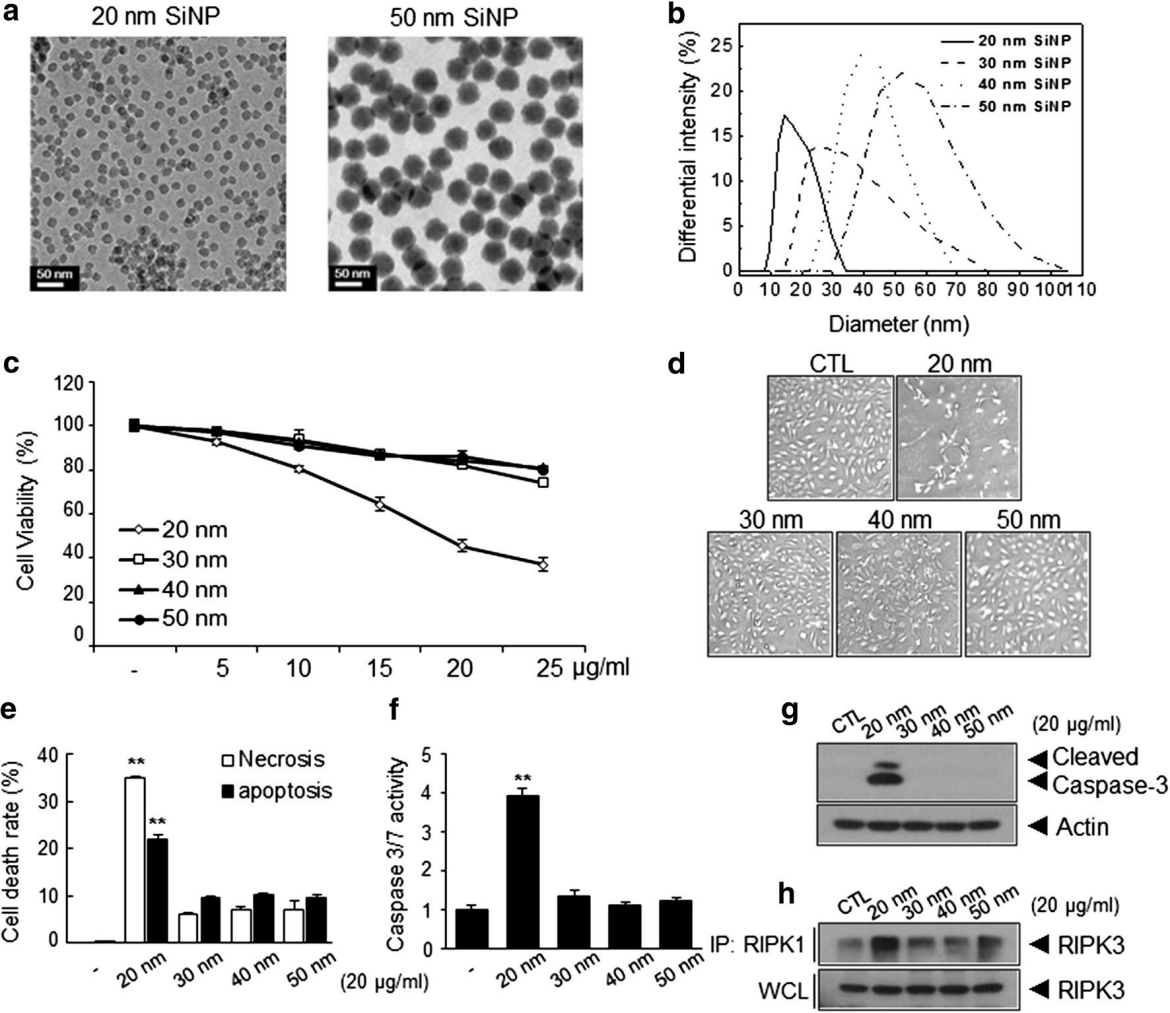
Characteristics and size-dependent cytotoxicity of silica nanoparticles (SiNPs). **a** TEM images of 20-nm and 50-nm SiNPs showing that both SiNPs were spherical and displayed monodispersity. **b** Analysis of DLS data from aqueous suspensions of SiNPs with different sizes ([SiNP] = 1 mg/mL). The size distribution was 18.7 ± 8.4, 30.2 ± 14.2, 37.8 ± 15.0, and 50.4 ± 21.1 nm. Size-dependent cytotoxicity of SiNPs in Human umbilical vein ECs (HUVECs). **c** Assessment of endothelial cell (EC) viability following treatment with different sizes of SiNPs. Human umbilical vein ECs (HUVECs) were treated with the indicated concentrations of the SiNPs for 24 h in low serum-containing condition and analyzed using crystal violet assay. **d** Morphologies of HUVECs following treatment with 20 μg/mL SiNPs for 24 h were showed using an optical microscopy, and **e** representative bar graph of the percentages of apoptotic and necrotic cells as determined by flow cytometric analysis (early apoptotic cell: annexin-V(+)/PI (−), late apoptotic cell: annexin-V(+)/PI (+), and necrotic cell: annexin-V(−)/PI (+); **p < 0.01 versus 30–50-nm SiNPs treated HUVECs). **f** Relative caspase 3/7 activity of HUVECs treated with 20 μg/mL SiNPs was quantified by Caspase-Glo 3/7 assay systems (**p < 0.01 versus 30–50-nm SiNPs treated HUVECs). Quantitative data are reported as means ± standard deviations. *p < 0.005 versus 30–50-nm SiNPs treated HUVECs. **g** Western blot analysis of caspase-3 activation in HUVECs following treatment with SiNPs for 24 h. **h** Interaction between RIPK1–RIPK3 was detected by immunoprecipitation (IP) and western blot analysis

We prepared SiNPs of various sizes using the modified method reported by Hartlen, utilizing a small variation of the solvent mixture ratio [23]. As shown in Fig. 1a and Additional file 1: Fig. S1, the morphology of ‘seed’ SiNPs was determined via transmission electron microscopy (TEM). Spherical SiNPs had a Feret diameter and monodispersity of 21.8 ± 0.6 nm, which was further increased to 31.4 ± 2.4, 42.9 ± 2.8, and 56.7 ± 1.5 via the regrowth procedure. Uncertainty was calculated in accordance with the Guide to Expression of Uncertainty in Measurement (GUM), revealing no overlap among SiNPs in terms of independent size distribution. Despite their monodispersity, some nanoparticles can aggregate in an aqueous environment. We additionally identified distinct size differences from the aqueous SiNP dispersions in phosphate-buffered saline (PBS) solution via the light scattering technique. As shown in Fig. 1b, the DLS data showed homogeneous dispersion and even a partial overlap between the SiNPs. However, when observing the effect of the serum on SiNPs, these dispersions changes broadly depending on the serum concentration (Additional file 1: Table S1). This insufficient serum stability may result from agglomeration in the SiNPs, leading to somewhat confusing results with respect to size-dependent cytotoxicity. In the meantime, several studies have been conducted in an environment that includes serum containing SiNPs sized 50 nm or more. However, there has been little research on the mechanisms of the toxicity of SiNPs that do not agglomerate in a size-dependent manner. Therefore, we decided to use the SiNPs dispersed in low serum-containing conditions (0.5%) for subsequent cytotoxicity experiments in this study.

### Induction of apoptosis and necrosis by SiNPs in ECs

In order to investigate the cytotoxicity of SiNPs of different sizes in ECs, human umbilical vein endothelial cells (HUVECs) were treated with various concentrations (5, 10, 15, 20, or 25 μg/mL) of SiNPs for 24 h, after which cellular viability was determined. SiNPs of 20-nm in size, but not those of 30 nm, 40 nm, and 50 nm in size, induced significant decreases in cellular viability in a dose-dependent manner (Fig. 1c, d), respectively, probably because the 30- and 40-nm SiNPs overlapped with the 50-nm SiNPs in an aqueous environment. When the size distribution of SiNPs was calculated from the PDI values, the size and standard deviation were 20.2 ± 8.1, 30.9 ± 10.5, 41.9 ± 7.2, and 51.9 ± 10.2 nm, respectively (${\text{PDI }} = \sqrt {{\text{standard deviation}}/{\text{mean diameter}}}$). The 20- and 50-nm SiNPs displayed distinct dispersions; therefore, we identified the severe cytotoxicity risk and mechanism underlying cell death with 20-nm SiNPs in comparison with those not overlapping the 50-nm SiNPs. To clarify whether these SiNP-mediated decreases in cellular viability resulted from the induction of apoptosis or necrosis, HUVECs were treated with SiNPs as indicated, stained with fluorescein isothiocyanate (FITC)-conjugated annexin V and propidium iodide (PI), and subjected to flow cytometric analysis (FACS). As shown in Fig. 1e, treatment with 20-nm SiNPs markedly induced apoptosis and necrosis in ECs, whereas other SiNPs did not induce significant levels of apoptosis or necrosis. Consistently, caspase-3/7 activation was predominantly increased in 20-nm SiNP-treated ECs (Fig. 1f, g). Necroptosis, which is an alternative form of programmed necrosis, is regulated by an oligomer complex comprising receptor interacting protein-1 and -3 (RIP-1 and RIP-3) [24]. The formation of the necroptosome, which is a key feature of programmed necrosis, may be investigated by co-immunoprecipitation of kinase RIP1 (RIPK1)/kinase RIP3 (RIPK3). Treatment of ECs with 20-nm SiNPs markedly increased RIPK3 activation by RIPK1 (Fig. 1h), indicating that 20-nm SiNPs additionally induce necrotic cell death in the ECs.

### Induction of ER stress-mediated apoptosis by SiNPs in ECs

**Fig. 2:**
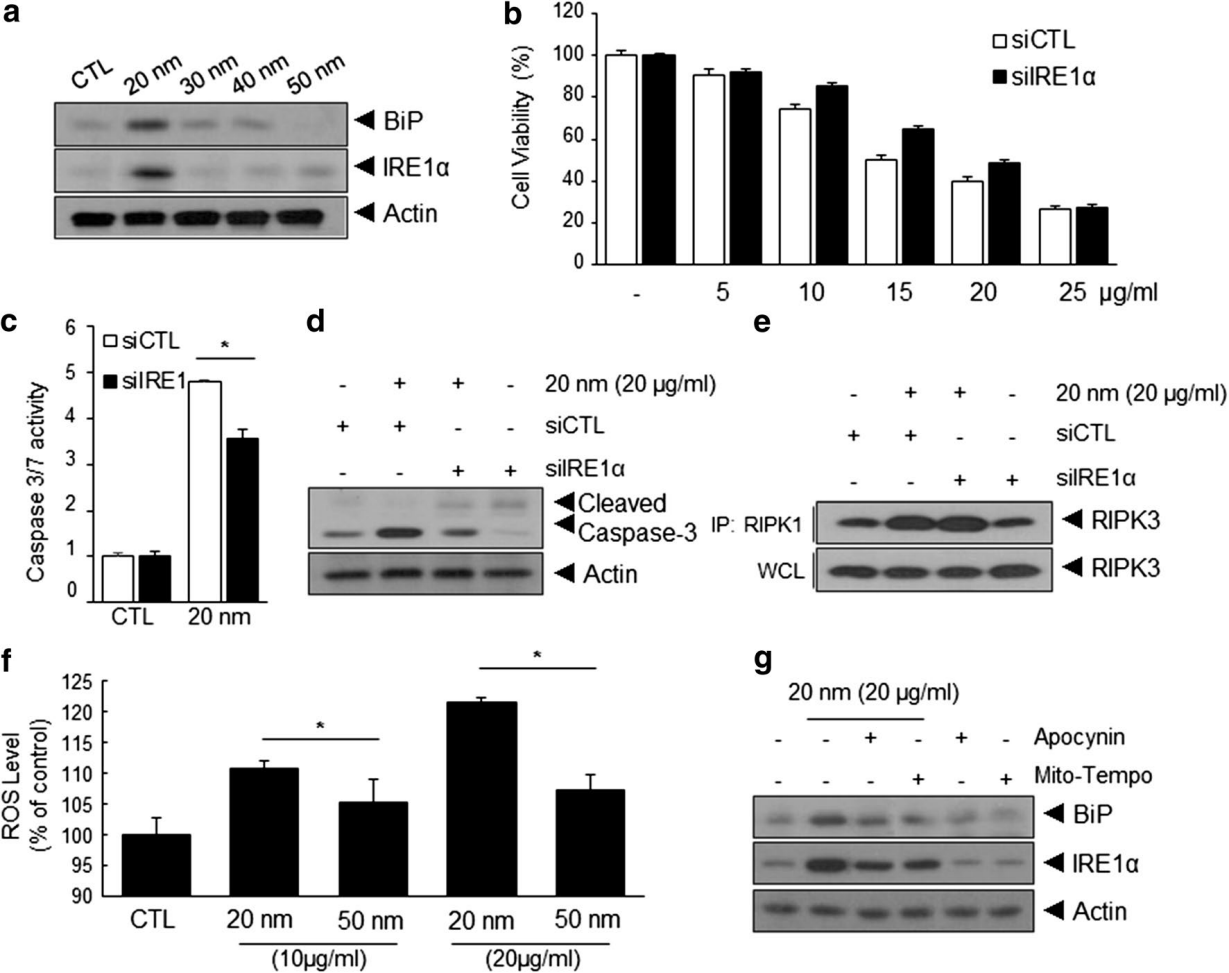
ROS/ER stress attenuated 20-nm SiNP-induced apoptosis cell death in HUVECs. **a** Western blot analysis of expression of Binding immunoglobulin Protein (BiP) and inositol-requiring kinase-1α (IRE1α) in HUVECs following treatment with different sizes of SiNPs for 24 h in low serum-containing condition. Effect of ER stress on SiNPs-induced apoptotic or necrotic cell death. HUVECs were transfected with IRE1α-targeted or control siRNA and subsequently treated with 20-nm SiNP for 24 h. **b** Dose-dependent cell viability, **c** relative caspase-3/7 activity, and **d**, **e** western blot analysis of caspase-3 activation and immunoprecipitation between RIPK1–RIPK3 in control or IRE1α siRNA-transfected HUVECs with SiNPs at 20 μg/mL for 24 h. Effect of an intracellular ROS inhibitors on SiNPs-induced ER stress in HUVECs. **f** Relative ROS level in HUVECs treated with the indicated sizes and concentration of SiNPs for 24 h. **g** Western blot analysis of expression of BiP and IRE1α in HUVECs were pretreated with 10 μM of Apocynin and Mito-Tempo for 30 min and subsequently treated with 20-nm SiNP for 5 h

ER stress plays a prominent role in cellular dysfunction [16]. To determine whether ER stress is involved in SiNP-induced apoptosis or necrosis in ECs, we first examined the effects of 20-nm SiNPs on the expression of binding immunoglobulin Protein (BiP) and inositol-requiring kinase-1α (IRE1α) as ER stress markers [25]. As shown in Fig. 2a, the expression of BiP and IRE1α was markedly increased in 20-nm SiNP-treated ECs, but not in cells treated with other SiNPs. To determine the role of IRE1α in 20-nm SiNP-mediated apoptosis and necrosis, HUVECs were treated with IRE1α-specific siRNA, and the effects of 20-nm SiNP treatment on apoptosis or necrosis were examined in these cells. Compared with that in control ECs, IRE1α knockdown significantly increased the viability of ECs following treatment with 20-nm SiNPs, as determined via the crystal violet assay, caspase-3/7 activity assay, and pro-caspase-3 cleavage (Fig. 2b–d). However, no significant differences in necrosis, as determined by the interaction between RIPK1 and RIPK3, were observed in siIRE1α-treated ECs (Fig. 2e), suggesting that 20-nm SiNPs induce apoptosis in an ER stress-dependent manner.

The core molecular machinery involved in the induction of ER stress has been studied in detail [26]. Among these, ROS are considered to be potent inducers of ER stress. Thus, we determined the intracellular ROS levels in ECs treated with SiNPs of 20-nm or 50-nm in size for 24 h. While intracellular ROS levels of ECs treated with 50-nm SiNPs increased slightly, treatment with 20-nm SiNPs led to a prominent increase in ROS levels (Fig. 2f). To determine whether 20-nm SiNP-induced ROS generation was involved in ER stress induction, HUVECs were pretreated with a mitochondria-targeted ROS scavenger, Mito-tempo, or a potential NADPH oxidase inhibitor, apocynin. As shown in Fig. 2g, the expression of BiP and IRE1α was suppressed following treatment with intracellular ROS inhibitors. Collectively, these results demonstrate that ROS-mediated ER stress is involved in 20-nm SiNP-mediated apoptotic cell death of EC, but not in necrotic cell death.

### Induction of autophagy in 20-nm SiNP-treated ECs

**Fig. 3:**
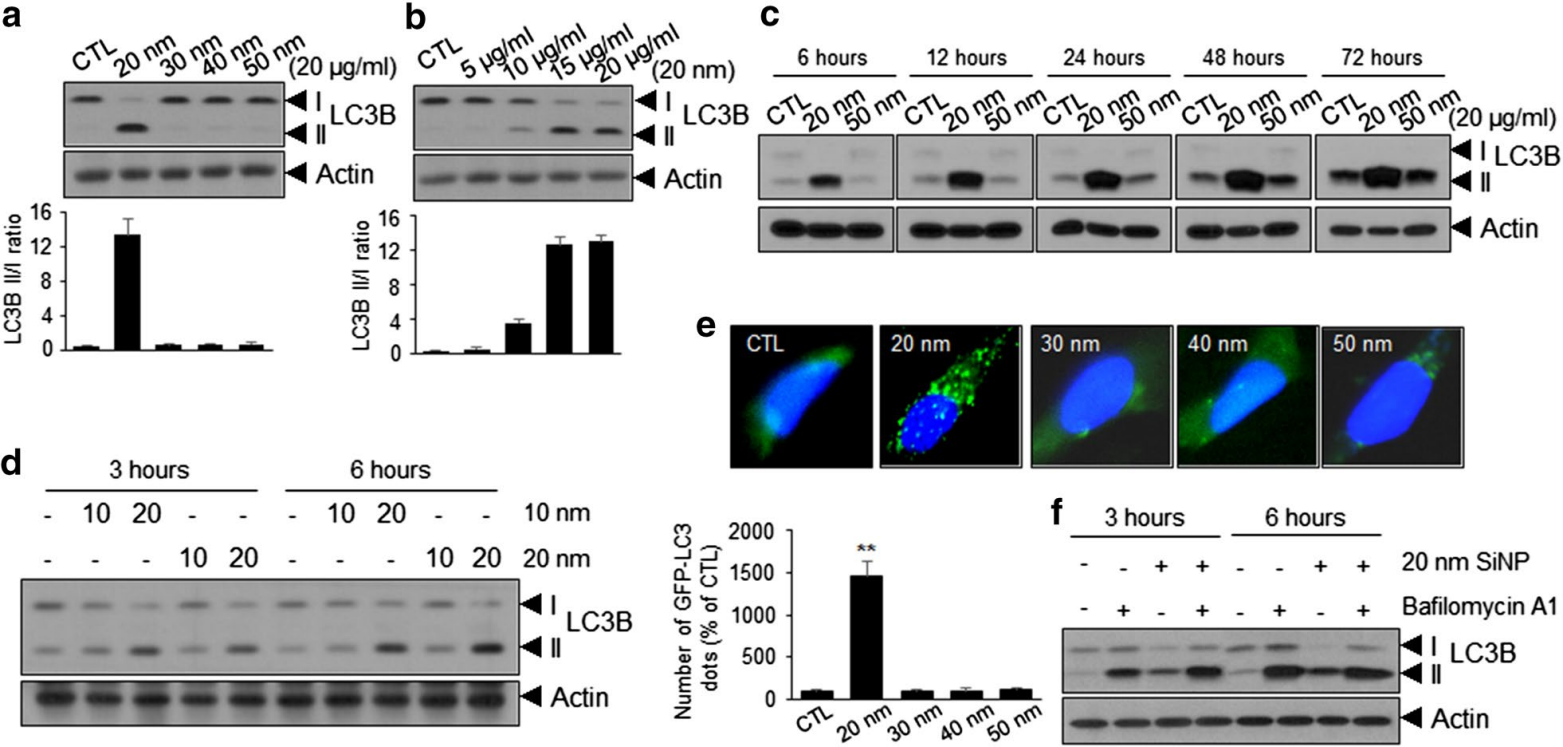
Induction of autophagy in 20-nm SiNP treated HUVECs. **a**–**d** Western blot analysis of microtubule-associated protein 1B-light chain 3 (LC3B)-I to LC3B-II conversion in HUVECs treated with the indicated sizes and concentration of SiNPs for 24 h in low serum-containing condition, and it also tested time-dependent analysis. Relative LC3-II to LC3-I ratios are indicated in the graph. Data were quantified using Image J software. **e** Representative images of green fluorescent protein (GFP)–LC3 punctae in HUVECs after treatment with SiNPs at 20 μg/mL for 12 h. Bar graph indicates the number of GFP–LC3 dots per transfected cells. Quantitative data are reported as means ± standard deviations. **p < 0.01 versus 30–50 nm SiNPs treated HUVECs. **f** Turnover assays for LC3 to determine the overall autophagic flux in HUVECs treated with 10 nM Bafilomycin A1 (lysosomal inhibitor) for 30 min and subsequently treated with 20-nm SiNP for 5 h. LC3-I to LC3-II conversion was detected by Western blot analysis

Numerous studies have reported the role of autophagy in the regulation of cell death; in particular, cross-talk between autophagy and apoptosis/necrosis has been reported [27, 28]. To clarify whether exposure to SiNPs triggers autophagy in ECs in a SiNP size-dependent manner, we examined the conversion of microtubule-associated protein 1A/1B-light chain 3 (LC3)-I to LC3-II, a hallmark of autophagy, in ECs treated with SiNPs of different sizes. Notably, treatment with 20-nm SiNPs increased autophagy in a dose- and time-dependent manner compared with treatment with other SiNPs (Fig. 3a–c). We attempted to determine whether autophagy is mainly induced in SiNPs below 20 nm in size: HUVECs were treated with 10-nm SiNPs for the same duration and at the same concentration. As shown in Fig. 3d, treatment with 10-nm SiNPs also induced LC3-II accumulation. Autophagy induction was further supported by the increased numbers of cytoplasmic punctae in ECs expressing green fluorescent protein (GFP)–LC3 fusion proteins (Fig. 3e). In contrast, a ubiquitous, diffuse pattern of cytosolic green fluorescence was observed in non-treated ECs, as well as in ECs treated with SiNPs of other sizes.

Although the accumulation of LC3-II or increased numbers of GFP-LC3 puncta indicate the induction of autophagy, this phenomenon may result from the interruption of autophagolysomal maturation or completion of autophagy [29]. Thus, we performed turnover assays for LC3 to determine whether the overall autophagic flux had been induced. As shown in Fig. 3f, ECs treated with 20-nm SiNPs in the presence of bafilomycin A1 (Baf A1), a lysosomal inhibitor, exhibited an increase in LC3-II accumulation, indicating that increased amounts of LC3 in autophagosomes had already been delivered to lysosomes for degradation. In addition, GFP-LC3 cleavage assay clearly showed cleavage GFP fragments resulting from GFP–LC3 degradation in autolysosomes (Additional file 1: Fig. S2A). Concurrently, the GFP-mRFP-LC3 construct was composed of GFP and red fluorescent protein (RFP) with different stability in acidic compartments; this construct is a valuable tool for identifying autophagosome maturation and autolysosome formation. pH-sensitive GFP is fluorescently quenched in the acidic environment of the autolysosome; however, RFP maintains detectable fluorescence intensity. Based on the difference in acidic stability between GFP and RFP, this construct enabled discrimination between autophagosomes and autolysosomes with yellow and red signals, respectively. ECs treated with 20-nm SiNPs showed both yellow and red puncta compared with the non-treated ECs, clearly indicating that the autophagic flux was increased in these cells (Additional file 1: Fig. S2B).

### Autophagy induced by 20-nm SiNPs is dependent on the PI3K/AKT/eNOS/nitric oxide signaling pathway, but independent of ROS or AMPK

**Fig. 4:**
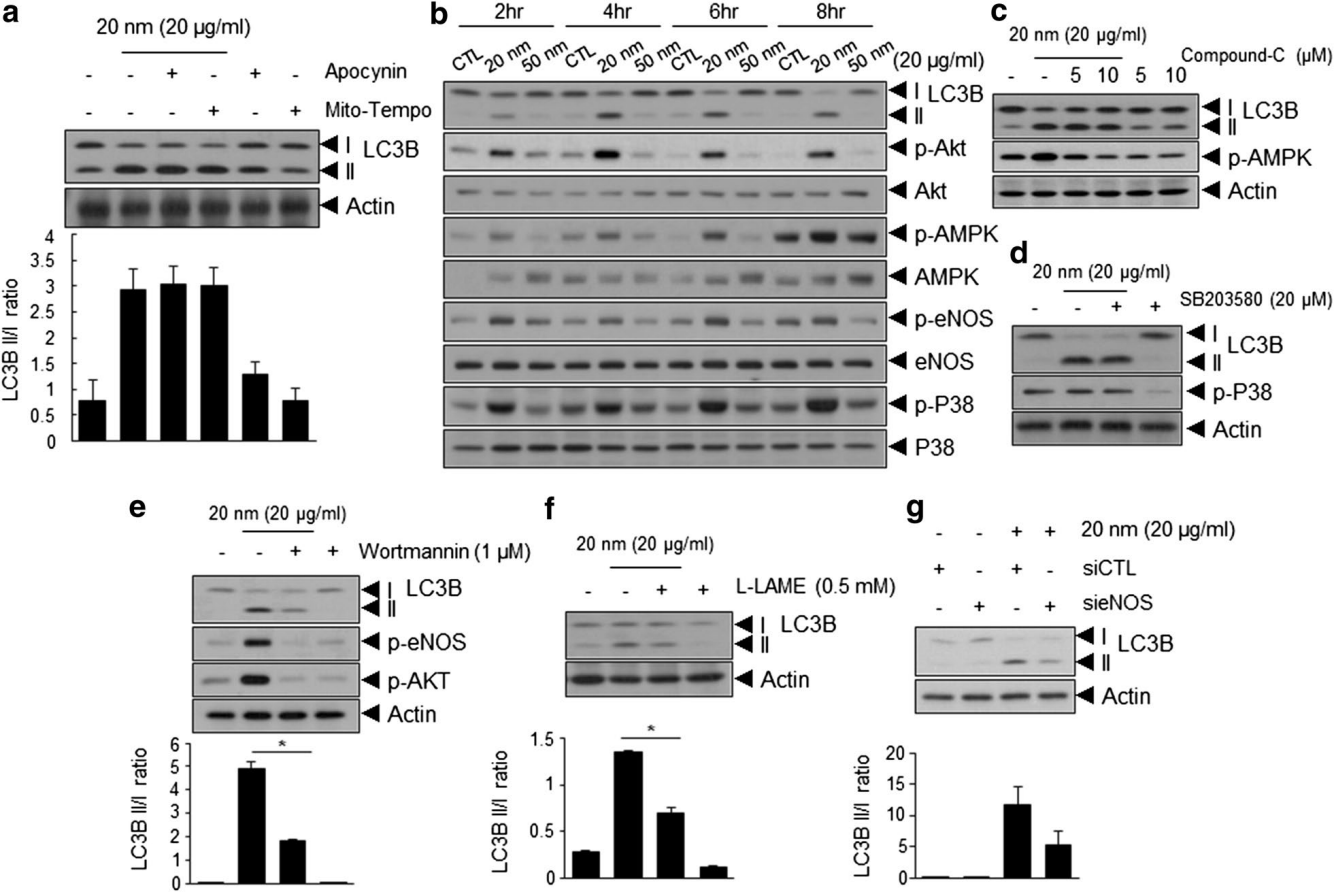
20-nm SiNP-induced autophagy depends on PI3K/AKT/eNOS/nitric oxide signaling pathway. **a** Western blot analysis of LC3B-I to LC3B-II conversion in HUVECs treated with 10 μM of Apocynin and Mito-Tempo for 30 min and subsequently treated with 20-nm SiNPs at 20 μg/mL for 5 h. Relative LC3-II to LC3-I ratios are indicated in the graph using Image J software. SiNPs-induced autophagy depends on PI3K/AKT or eNOS signaling pathway activation, but independent of ROS or AMPK. **b** Western blot analysis of LC3B-I to LC3B-II conversion and and AKT, AMP-activated protein kinase (AMPK), endothelial NO-synthase (eNOS), and p38 phosphorylation. HUVECs were treated with 20- and 50-nm of SiNPs for the indicated times at 20 μg/mL. **c**, **d** Western blot analysis of LC3-I to LC3-II conversion and AMPK phosphorylation or p38 phosphorylation in HUVECs treated with indicated concentrations of Compound-C and SB203580 for 30 min and subsequently treated with 20-nm SiNPs at 20 μg/mL for 5 h, respectively. **e**–**g** Western blot analysis of the LC3-I to LC3-II conversion following treatment with PI3K inhibitor (Wortmannin), eNOS inhibitor (L-LAME), and eNOS siRNA, respectively. Relative LC3-II to LC3-I ratios are quantified by Image J software

We investigated the signaling pathways involved in 20-nm SiNP-mediated autophagy induction in ECs. Among the various signaling pathways, ROS is considered a crucial regulator of autophagy [30]. To determine whether 20-nm SiNP-induced ROS generation was involved in autophagy induction, HUVECs were pretreated with Mito-tempo or apocynin. As shown in Fig. 4a, this did not appear to affect the induction of autophagy, as treatment with Mito-tempo or apocynin did not impair the accumulation of LC3-II in 20-nm SiNP-treated HUVECs. Furthermore, we examined the effects of SiNPs on the activation of signaling molecules associated with autophagy induction in ECs. As shown in Fig. 4b and Additional file 1: Fig. S3, treatment with 20-nm SiNPs markedly induced the phosphorylation of p38, AMPK, AKT, and eNOS, but not that of Src, ERK, JNK, p53, and mTOR. Thus, we treated ECs with inhibitors of the 20-nm SiNP-activated signaling molecules to elucidate whether they are involved in the induction of autophagy. Despite changes in the phosphorylation of AMPK and P38, no significant differences were observed in 20-nm SiNP-treated ECs in the presence of Compound-C, an AMPK inhibitor, or SB203580, a p38 inhibitor (Fig. 4c, d). In contrast, inhibition of the PI3K/AKT/eNOS signaling pathway by treatment with Wortmannin, a PI3K inhibitor, resulted in a significant decrease in 20-nm SiNP-induced LC3-II accumulation (Fig. 4e). Consistently, 20-nm SiNP-induced LC3-II accumulation was also significantly attenuated by treatment with the eNOS inhibitor, L-LAME (Fig. 4f). Similarly, the transfection of ECs with eNOS-specific siRNA after treatment with 20-nm SiNPs significantly inhibited the accumulation of LC3-II (Fig. 4g), indicating that 20-nm SiNP-induced autophagy is mediated by the PI3K/AKT/eNOS signaling axis.

### Inhibition of autophagy attenuates 20-nm SiNP-induced necrotic cell death, but not apoptotic cell death, in ECs

**Fig. 5:**
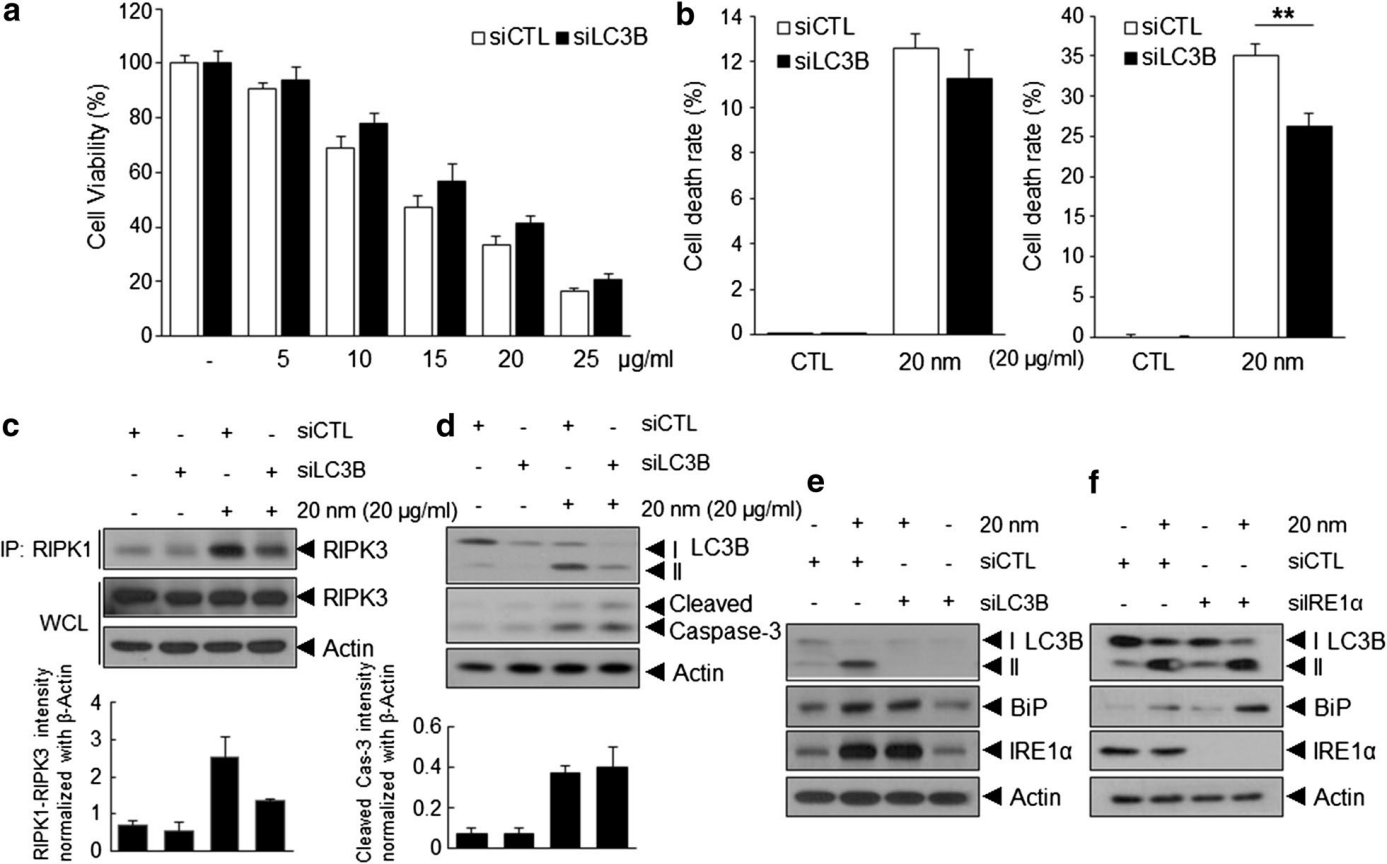
Induction of autophagy attenuated 20-nm SiNP-induced necrotic cell death in HUVECs. HUVECs were transfected with LC3B-targeted or control siRNA and subsequently treated with the indicated concentrations of the 20-nm SiNP for 24 h in low serum-containing condition. **a** Dose-dependent cell viability was determined using the crystal violet assay. **b** Representative bar graph of the percentages of apoptotic and necrotic cells was quantified by flow cytometric analysis. **c**, **d** Western blot analysis of immunoprecipitation between RIPK1–RIPK3 and caspase-3 activation in the transfected cells. **e**, **f** Western blot analysis of the BiP, IRE1α, and LC3-I to LC3-II conversion in HUVECs were transfected with LC3B or IRE1α siRNA and subsequently treated with the indicated concentrations of the 20-nm SiNP for 24 h in low serum-containing condition

To determine the role of autophagy in 20-nm SiNP-induced apoptosis or necrosis, HUVECs were treated with 20-nm SiNPs following transfection with LC3-specific siRNA. LC3-specific siRNA treatment decreased 20-nm SiNP-induced autophagy, as determined by reduced LC3-II accumulation in these cells and resulted in a significantly increased viability of ECs after treatment with 20-nm SiNP as determined via the crystal violet assay (Fig. 5a). To clarify whether 20-nm SiNP-induced autophagy is involved in apoptotic or necrotic cell death, HUVECs were stained with FITC-conjugated annexin V and PI and subjected to FACS. Interestingly, LC3-specific siRNA treatment significantly decreased necrotic cell death, while no significant differences were observed in terms of the levels of apoptotic cell death (Fig. 5b). Consistent with these results, interaction between RIPK1 and RIPK3 was substantially decreased, whereas no significant change in pro-caspase-3 cleavage was observed in 20-nm SiNP-treated ECs (Fig. 5c, d), indicating that 20-nm SiNP-induced autophagy is involved in necrotic cell death, but not in apoptotic cell death.

**Fig. 6:**
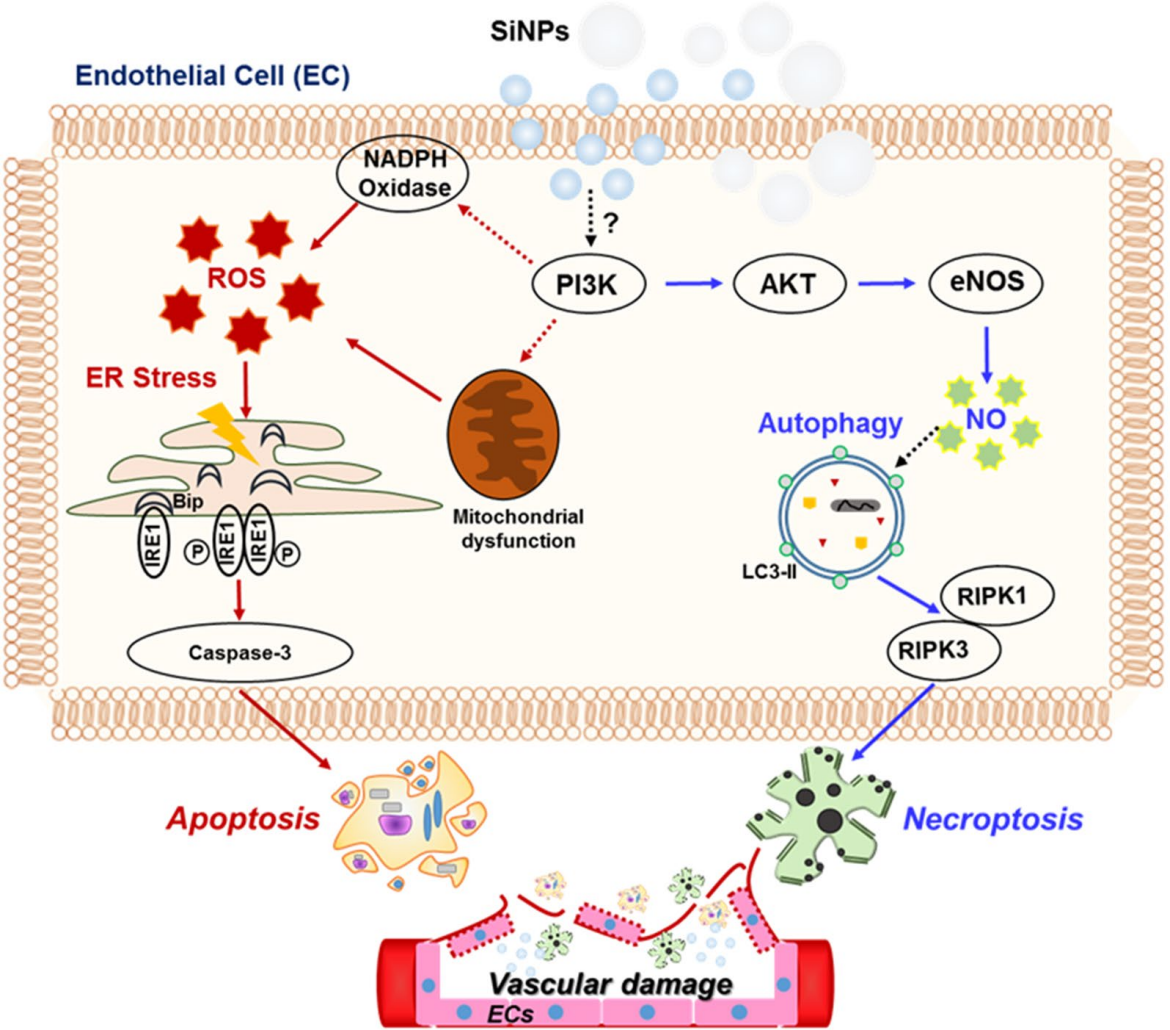
Overview of SiNPs-induced apoptotic and necrotic cell death pathways independently in HUVECs. The 20-nm SiNP exposed to the membrane of endothelial cells, leading to intracellular ROS level. NADPH oxidase (NOX), a non-mitochondrial source of ROS increase, generates superoxide anions through oxygen reduction mediated by the electron donor NADPH. NOX-derived ROS contribute ER stress and activate unfolded protein response (UPR), resulting that IRE1α dissociated from BiP responses the unfolded proteins. Subsequently, the resultant trans-autophosphorylation induce apoptotic cell death. In addition, 20-nm SiNP induce autophagy activation, independent of ROS, via PI3K/AKT/eNOS/nitric oxide signaling pathway. Under the induction of autophagy, RIP1 interacts with RIP3 to form complex Iib, which is involved in necroptosis. As a result, autophagy induced by 20-nm SiNP causes necrotic cell death

Previous studies have reported that ER stress and autophagic activity occur simultaneously [31]. We assessed whether autophagy is associated with ER stress following 20-nm SiNP exposure in ECs. Unexpectedly, the transfection of ECs with LC3 or IRE1α-specific siRNA did not alter the expression of BiP and IRE1α or LC3-II accumulation in 20-nm SiNP-treated ECs, respectively (Fig. 5e, f), suggesting that 20-nm SiNP-induced autophagy or ER stress is independently involved in EC viability by inducing apoptosis or necrosis (Fig. 6).

## Discussion

SiNPs reportedly constitute a versatile backbone platform for biomedical applications owing to carrier stability, tunable pore sizes, and drug loading and controlled release. Recent studies have focused on coating or surface incorporation of biocompatible materials, such as lipids and polymers, to improve the drug loading efficiency and carrier stability. SiNPs are clearly biodegradable and safe in extensive animal studies; however these results have been mostly obtained with approximately 100-nm nanoparticles [32]. Recent Stöber methods, primarily used to synthesize SiNPs, can produce sufficiently smaller nanoparticles, with better biological effects being expected. However, cytotoxicity would similarly be driven with a stronger or new pathway, depending on the size of the SiNPs or their increased surface area. Assessment of size-dependent cytotoxicity of sub-100-nm particles is important to establish safe design criteria for SiNPs. Cell viability reportedly deteriorates with a reduction in SiNP size. Unfortunately, these results were evaluated with various SiNPs ranging 20–100 nm in size, to prevent overlaps in their size distribution. Few studies have described the cellular response of SiNPs of below 50 nm in size at low concentration levels, particularly below 25 μg/mL. Moreover, most of these studies have failed to elucidate the detailed molecular mechanism underlying cytotoxic effects associated with SiNP size on cells. Eventually, to date, no clear guidelines regarding the acceptable size of SiNPs are available in terms of biological safety.

The biomedical applications of SiNPs requires their intravenous administration. As a result, ECs, which line blood vessels, form the primary site of contact with SiNPs. Despite this, the current understanding of the effects of SiNPs on the vasculature is very limited. Moreover, clear assessment of the size-dependent toxic effects on the ECs could not be performed, as blood proteins, including serum proteins, may lead to the formation of SiNP agglomerates of hundreds of nanometers in size. In this study, to explore the size-dependent cellular responses of SiNPs in ECs, four sets of SiNPs of different size were prepared, and cells were treated with these nanoparticles under overlap- or agglomerate-free conditions. The present study is the first to demonstrate ER stress-mediated apoptotic cell death and autophagy-induced necrotic cell death in ECs in response to SiNPs of different sizes. The 20-nm SiNPs, but not SiNPs of other sizes, induced significant cellular apoptosis via the caspase-cascade pathway. RIPK1/RIPK3 signaling was also upregulated in the 20-nm SiNPs-treated ECs compared with that in ECs treated with other SiNPs, indicating the occurrence of programmed necrosis. Moreover, this study provides a strategy for overcoming SiNP-induced cytotoxicity by providing insights into the mechanism underlying SiNP-induced ER stress and autophagy in ECs.

The ER ensures an appropriate folding environment for newly synthesized and secreted proteins [33]. ER dysfunction or ER stress is one of the most important factors affecting cell survival; cells undergo programmed cell death under excessive ER stress [34]. The unfolded protein response (UPR) is mediated initially by three major ER membrane transducers, which serve as stress-sensing proteins: PRKR-like ER kinase (PERK), activating transcription factor 6 (ATF-6), and IRE1α [35]. Under unstressed conditions, the luminal domains of these proteins remain inactive, forming a stable complex with the ER resident chaperone BiP [25]. When the unfolded proteins accumulate in the ER, the BiP dissociates from these complexes to refold these proteins. In addition, IRE1α, upon dissociation from BiP, senses and responds to the unfolded proteins via oligomerization of its luminal domains, leading to trans-autophosphorylation and subsequent RNase activation [36]. Although the induction of these proteins following SiNP exposure has been reported in various cell lines, their expression and the mechanism underlying SiNP-mediated ER stress-induced apoptosis have not fully been elucidated in ECs. Our results show that intracellular ROS levels in SiNP-treated ECs increased in a particle size-dependent manner (Fig. 2f). Increased ROS levels stimulated the expression of BiP and IRE1α protein, leading to apoptotic cell death (Fig. 2g). Interestingly, SiNP-mediated ER stress induced apoptotic cell death rather than necrotic cell death. Accordingly, the inhibition of ER stress by IRE1α knockdown reduced caspase activation but not necroptosome formation (Fig. 2c–f).

Recently, nanotoxicological studies in different types of ECs have reported that SiNPs can modulate autophagy, which is a major contributor to SiNP-induced cytotoxicity and dysfunction [37–40]. However, these studies report that autophagy is induced by SiNPs sized 50 nm or greater at a relatively high concentration. Moreover, most of these studies analyzed the effect of SiNPs under high serum-containing conditions. Small SiNPs are not suitable for analysis of cytotoxic effects under high serum-containing conditions, as SiNPs undergo agglomeration in serum; therefore, absorption of SiNPs by the cells is relatively reduced [40] In this regard, our study is the first to demonstrate that autophagy is induced by SiNPs of sizes smaller than 50 nm at low serum concentrations (0.5%). We observed that only SiNPs of 20 nm in size or smaller, but not those of 30 nm, 40 nm, or 50 nm in size, induced autophagy in ECs at low serum-containing conditions (Fig. 3a–c). Interestingly, blockade of autophagy with LC3-specific siRNAs significantly reduced necrotic cell death rather than apoptotic cell death (Fig. 5). Collectively, these results suggest that the type of EC death induced by SiNPs varies depending on the serum concentration and of SiNP size.

This study provides insights into the mechanism by which 20-nm SiNPs induce autophagy. MAPK signaling, p53-mediated AMPK/mTOR signaling, and ROS have been previously implicated in the regulation of cell death and autophagy [29]. Recent reports show that SiNPs induces autophagy via ROS-mediated MAPK/Bcl-2 and PI3K/Akt/mTOR signaling in ECs [41, 42]. However, in the present study, no significant differences between 20-nm SiNP- and 50-nm SiNP-treated ECs were observed in terms of the phosphorylation of ERK, JNK, or mTOR and the expression of p53 (Additional file 1: Fig. S3). Moreover, the scavenging of ROS or pharmacological inhibition of NADPH oxidase (Fig. 4a), p38, and AMPK had no significant effects on 20-nm SiNP-mediated autophagy induction (Fig. 4c, d). Interestingly, the pharmacological inhibition of PI3K/AKT/eNOS signaling or siRNA knockdown of eNOS significantly decreased LC3-II accumulation following treatment of ECs with 20-nm SiNPs. These results suggest that SiNP-induced autophagic signaling pathways differ depending on the concentration of serum and the size of SiNPs; accordingly, the mechanisms underlying cytotoxicity (apoptosis or necrosis) may be different in ECs exposed to SiNPs.

Numerous studies have reported that the size and shape of nanoparticles affect their intracellular uptake [43]. The 20-nm SiNPs, which are significantly smaller than the 50-nm SiNPs, are predicted to accumulate more towards the interior of cells during equal treatment. As shown in Additional file 1: Fig. S4A, cellular uptake of FITC-labeled 20-nm and 50-nm SiNPs was assessed via FACS analysis. When the SiNPs were treated at equal concentrations, we observed stronger FITC fluorescence intensities in cells treated with 20-nm SiNPs than with 50-nm SiNPs. Prominent intracellular uptake of 20-nm SiNPs primarily causes cell death more strongly than SiNPs of other sizes. This result is consistent with the considerable dose-dependent cytotoxicity observed with 20-nm SiNPs. In addition, we broadened the treatment range of SiNPs to obtain IC50 values for 50-nm SiNPs amounting to 78.2 μg/mL (Additional file 1: Fig. S4B). An increase in the number of 50-nm SiNPs treated with cells increased the accumulation of intracellular SiNPs. However, although 50-nm SiNPs were treated at the same IC50 values, they did not approach levels of intracellular accumulation obtained with 20-nm SiNPs (Additional file 1: Fig. S4C). These observations suggest that the extent of intracellular accumulation alone cannot completely interpret the cytotoxic effects of SiNPs.

To determine whether 50-nm SiNP-mediated cell death at IC50 resulted from the induction of apoptosis or necrosis, HUVECs were treated with 50-nm SiNP as indicated, stained with FITC-conjugated annexin V and propidium iodide (PI), and subjected to flow cytometric analysis. Unlike 20-nm SiNPs, 50-nm SiNPs predominantly induced apoptotic rather than necrotic cell death (Additional file 1: Fig. S5A). Furthermore, we assessed the levels of ER stress markers including BiP and IRE1α in 50-nm SiNP-treated HUVECs to evaluate the potential involvement of ER stress in 50-nm SiNP-mediated apoptotic cell death. As shown in Additional file 1: Fig. S5B, BiP or IRE1 was not significantly induced in 50-nm SiNP-treated ECs. Moreover, neither increased interactions between RIPK1 and RIPK3 nor autophagy induction were observed in 50-nm SiNP-treated ECs (Additional file 1: Fig. S5C, D). Together, these results indicate that 20-nm SiNPs result in EC cytotoxicity through distinct cell death pathways compared to other SiNPs.

## Conclusions

To our knowledge, this study is the first to provide experimental data on the biological effects associated with the size of SiNPs under aggregation-free conditions such as at low serum concentrations in ECs. Furthermore, the results indicate that 20-nm SiNPs induce EC cell death through the induction of ER stress and autophagy-independent signaling mechanisms. As shown in Fig. 6, 20-nm SiNPs induce ROS/ER stress-mediated apoptotic cell death and autophagy-mediated necrotic cell death through the PI3K/AKT/eNOS signaling axis. Although further investigations in vivo are necessary to confirm that SiNPs with a diameter < 20 nm pose greater risks to cells in terms of cytotoxic effects, the present data provide an enhanced understanding of the mechanism underlying SiNP size-dependent cytotoxicity in the vasculature. The present findings should inform the formulation of safety guidelines for biomedical applications of SiNPs.

## Methods

### Preparation and characterization of SiNPs with different sizes

Four kinds of SiNPs were fabricated using modified Stöber methods as previously described [44]. Briefly, 350 mL of 8.2 mM aqueous l-arginine (Sigma-Aldrich) was prepared in in a 500-mL round-bottom flask, and it was warmed to 50 °C. The mixture of tetraethylorthosilicate (TEOS, 98%, ACROS) and cyclohexane (99%, Samchun Chemicals) was added (TEOS:cyclohexane = 3:2) to the solution and vigorously stirred for 24 h, resulting in the 20-nm SiNPs. The SiNPs sized 30 nm were fabricated via almost same method with 20-nm SiNPs. 6 mL of the mixture of TEOS and cyclohexane (1:0.8) was added to 41.4 mL of 2.2 mM aqueous l-arginine and the temperature was kept overnight at 70 °C. To obtain 40 and 50 nm sized SiNPs, 5 mL of TEOS was added to the 30-nm SiNPs reaction solution at 24-h intervals for serial regrowth. These resultant SiNPs suspensions were collected by using a separatory funnel to isolate from organic layer. TEM specimens were prepared by placing the droplets of SiNPs suspension onto 200-mesh copper grids. The grids are allowed to completely dry before the measurement. The TEM images were obtained by JEOL, JEM-ARM200F at 200 kV of accelerating voltage. The uncertainty was calculated according to Guide to Expression of Uncertainty in Measurement (GUM), and the result was the measurement of the size of over 150 SiNPs. The hydrodynamic sizes of SNPs in aqueous suspensions was determined using a Nano ZS Zetasizer (Malvern, Worcestershire, UK). All synthesis and measurements processes were performed in deionized water (Milipore-Q water, 18.2 MΩ cm).

### Cell culture

HUVECs were isolated from human umbilical cord veins by collagenase treatment as described previously [45]. The HUVECs grown on 1% gelatin-coated tissue-culture dish in M199 media (Welgene) with 20% fetal bovine serum (FBS, HyClone), 1× Antibiotic–Antimycotic (Gibco), 3 ng/mL of basic fibroblast growth factor (Millipore), and 5 units/mL heparin (Sigma-Aldrich) at 37 °C under a 95% humidity and 5% (v/v) mixture of air and CO_2_. The cells from passages 2–7 were subsequently used in experiments and it was starved for 3 h with M199 media with 0.5% FBS before treatment of SiNPs.

### Reagents and antibodies

Bafilomycin A1, Apocynin, Mito-Tempo, Compound-C, Wortmannin, L-LAME were purchased from Sigma-Aldrich. The siRNAs for human IRE1α, LC3B, and eNOS were purchased from Santa Cruz Biotechnology. Control siRNA was purchased from Bioneer (Daejeon, Korea). SB203580 was obtained from Calbiochem. Fluo-4/NM was purchased from Molecular Probes. The primary antibodies used as follows. Anti-Cleaved Caspase-3, anti-BiP, anti-IRE1α, anti-phospho-Akt, anti-Akt, anti-phospho-AMPK, anti-AMPK, anti-phospho-P38, anti-P38, anti-phospho-ERK, anti-ERK, anti-phospho-mTOR, anti-mTOR, anti-phospho-Src, anti-Src, anti-P53 (Cell Signaling Technology); anti-Actin (Abclon); anti-RIPK-1, anti-RIPK-3, anti-phospho-eNOS, anti-eNOS (BD Bioscience); anti-LC3B (Sigma-Aldrich); anti-GFP, anti-phospho-JNK, anti-JNK (Santa Cruz); goat anti-rabbit (Pierce Biotechnology); goat anti-mouse (Abclon).

### Cytotoxicity assay

The HUVECs were seeded (1 × 10^5^ cells/well) in a 12-well plate and incubated for 24 h at 37 °C. After serum starvation, cells were washed using phosphate-buffered saline (PBS), and media containing SiNPs was added at different concentrations (5, 10, 15, 20, 25 μg/mL) for 24 h. Thereafter medium was discarded carefully, the cells were fixed with 3% formaldehyde (Sigma-Aldrich) and stained by 0.5% crystal violet (Sigma-Aldrich) staining solution with 30% ethanol (Merck) for 30 min at room temperature. After 30 min, crystal violet staining solution was discarded, and the wells were washed with distilled water twice and then dried. Thereafter 1% Sodium dodecyl sulfate (SDS, Sigma-Aldrich) solution was added into each well. The absorbance at 550 nm was measured with a microplate reader.

### Apoptosis and necrosis analysis

The HUVECs were treated with 20 μg/mL SiNP for 12 h and caspase 3/7 activity was measured with Caspase-Glo 3/7 assay systems (Promega) in accordance with the manufacturer’s instructions. In brief, equal volume of Caspase-Glo 3/7 reagent was added to lysates, followed by incubation at room temperature for 3 h. The luminescence of each sample was measured using microplate luminometer. Apoptosis and/or necrosis of the HUVECs was determined by the FITC-Annexin-V and propidium iodide (PI) assay. HUVECs were seeded in 60 mm-plates for 24 h and SiNPs were treated for 12 h in low serum-containing condition. Thereafter, cells were washed with PBS, harvested, and double-stained with FITC-Annexin-V and PI (BD bioscience) in accordance with the manufacturer’s instructions. Cells were analyzed by flow cytometry using FACSCalibur (BD bioscience) and data analysis was performed using FlowJo software.

### Measurement of reactive oxygen species

The HUVECs were seeded (1 × 10^4^ cells/well) in a 96-well dark plate and incubated for 24 h at 37 °C. The cells were treated 20-and 50-nm SiNPs at 10 or 20 μg/mL for 6 h, respectively, and stained by H_2_DCFDA (Molecular probes) solution for 30 min at room temperature. Then, It was added general oxidative stress indicator, H_2_DCFDA (Molecular probes) solution for 30 min. After the solution was discarded carefully, the wells were filled with PBS buffer and immediately DCF fluorescence signals were detected using a flow cytometer (Ex/Em = 485/535). Data analysis was carried out using WinMDI2.9 software.

### Western blot analysis and immunoprecipitation

For western blotting of HUVECs treated with SiNP and/or desired inhibitors, the cells were harvested under nondenaturing conditions, the media were removed, and cells were rinsed with ice-cold PBS. Whole-cell protein extracts were resuspended in RIPA lysis buffer containing 50 mM Tris/Cl, pH 7.6, 150 mM NaCl, 1 mM EDTA, 0.5% Na-deoxycholate, 1% Triton X-100, 50 mM β-glycerophosphate, 50 mM NaF, 1 mM Na_3_VO_4_, 1 mM PMSF, 10 μg/mL Aprotinin, 10 μg/mL Leupeptin, and 0.5% NP-40. Cell lysates were resuspended 6X sample buffer, heated 95 °C and centrifuged. The samples were subjected to SDS-PAGE, and the proteins were transferred to polyvinylidene fluoride (PVDF, Millopore) membranes. The membranes were incubated with primary antibodies at 4 °C overnight. Thereafter membranes were incubated with species-specific horseradish peroxidase (HRP)-conjugated secondary antibodies. The immunoreactive bands were visualized with a chemiluminescent substrate (GE Life Sciences).

For immunoprecipitation, cell lysates were prepared by NP-40 cell lysis buffer (50 mM Tris–Cl, 150 mM NaCl, 50 mM β-glycerophosphate, 50 mM NaF, 1 mM Na_3_VO_4_, 1 mM PMSF, 10 μg/mL Aprotinin, 10 μg/mL Leupeptin, and 1% NP-40). The cell lysates were pre-cleared with protein G-agarose beads (Millipore) at 4 °C for 1 h and incubated overnight at 4 °C with primary antibody. Thereafter, protein G-agarose was added and incubated for 2 h, and the sample was washed with NP-40 lysis buffer and resuspended in 2× loading buffer, heated 95 °C, and centrifuged.

### Quantification of GFP-LC3 puncta formation and mRFP-GFP-LC3 color change

Autophagosome formation was determined on the basis of accumulation GFP-LC3 or RFP-LC3 punctate foci in HUVECs, 1 μg of GFP-LC3 or mRFP-GFP-LC3 expression vector was transfected into HUVECs using Neon transfection system (Life Technology) in accordance with the manufacturer’s instructions. After incubation of 12 h, the transfected cells were treated with 20-nm SiNP for 6 h and then fixed with 3.7% formaldehyde. Cell images were obtained via fluorescence microscopy and quantified with the Metamorph 7.1 program.

### siRNA transfection and transduction

For knockdown experiments using RNAi, 100 pmol of siRNA was transfected into HUVECs via Neon transfection system. Briefly, cells were washed with no-serum M199 media, resuspended in 100 μL R buffer (provided in the Neon kit), and mixed with 100 pmol of siRNA. After electroporation, the cells were subjected to SiNP treatment at 20 μg/mL for 12 h.

### Statistical analysis

Quantitative data are expressed as mean ± standard deviation values based on at least triplicate observations from three independent experiments. Differences between groups were analyzed using Student’s t test. *p-value of < 0.05 and **p-value of < 0.01 were considered statistically significant.

## Additional file

**Additional file 1: Table S1.** Size distribution of SiNPs with 20 and 50 nm sizes at 1 mg/mL in various condition containing serum (0.5, 1.0, and 10 %; v/v). **Figure S1.** Morphology and size distribution of SiNPs. **Figure S2.** GFP fragments were degraded from GFP–LC3 in autolysosomes. **Figure S3.** 20-nm SiNP-induced autophagy is independent of JNK or p53-mediated AMPK/mTOR signaling pathways. **Figure S4.** Size and dose-dependent cellular uptake of SiNPs. **Figure S5.** Size-dependent toxic mechanisms of SiNPs.

## Abbreviations

SiNPs: silica nanoparticles
ECs: human endothelial cells
ROS: reactive oxygen species
VWF: von Willebrand factor
ER: endoplasmic reticulum
TEM: transmit electron microscopy
PBS: phosphate-buffered saline
HUVECs: human umbilical vein endothelial cells
FITC: fluorescein isothiocyanate
RIP-1: receptor interacting protein-1
RIP-3: receptor interacting protein-3
RIPK1: kinase RIP1
RIPK3: kinase RIP3
BiP: binding immunoglobulin Protein
IRE1α: inositol-requiring kinase-1α
NADPH: dihydronicotinamide-adenine dinucleotide phosphate
LC3: microtubule-associated protein 1A/1B-light chain 3
GFP: green fluorescent protein
BafA1: bafilomycin A1
RFP: red fluorescent protein
AMPK: AMP-activated protein kinase
eNOS: endothelial nitric oxide synthase
ERK: extracellular signal-regulated kinase,
JNK: c-Jun N-terminal kinases
mTOR: mammalian target of rapamycin
UPR: unfolded protein response
PERK: PRKR-like ER kinase
ATF-6: activating transcription factor 6
MAPK: mitogen-activated protein kinase
Bcl-2: B cell lymphoma-2
TEOS: tetraethylorthosilicate
FBS: fetal bovine serum
SDS: sodium dodecyl sulfate
PI: propidium iodide
RIPA: radioimmunoprecipitation assay
EDTA: ethylenediaminetetraacetic acid
PMSF: phenylmethylsulfonyl fluoride
PVDF: polyvinylidene fluoride
HRP: horseradish peroxidase
