# Supplementary material for: Two distinct cellular pathways leading to endothelial cell cytotoxicity by silica nanoparticle size
Source: J Nanobiotechnology. 2019 Feb 5;17:24. doi: 10.1186/s12951-019-0456-4 (PMC6362579; doi:10.1186/s12951-019-0456-4)
Supplement: Supplementary file 1 — Additional file 1: Table S1. Size distribution of SiNPs with 20 and 50 nm sizes at 1 mg/mL in various condition containing serum (0.5, 1.0, and 10 %; v/v). Figure S1. Morphology and size distribution of SiNPs. Figure S2. GFP fragments were degraded from GFP–LC3 in autolysosomes. Figure S3. 20-nm SiNP-induced autophagy is independent of JNK or p53-mediated AMPK/mTOR signaling pathways. Figure S4. Size and dose-dependent cellular uptake of SiNPs. Figure S5. Size-dependent toxic mechanisms of SiNPs. [file 12951_2019_456_MOESM1_ESM.docx]

Two distinct cellular pathways leading to endothelial cell cytotoxicity by silica nanoparticle size

Kyungmin Lee ^1, †^, Jangwook Lee ^1, †^, Minjeong Kwak ^2^, Young-Lai Cho ^3^, Byungtae Hwang ^1^, Min Ji Cho ^1,4^, Na Geum Lee ^1,4^, Jongjin Park ^1,4^, Sang-Hyun Lee ^1^, Jong-Gil Park ^1^, Yeon-Gu Kim ^1^, Jang-Seong Kim ^1^, Tae-Su Han ^1^, Hyun-Soo Cho ^1^, [Young-Jun Park](http://www.sciencedirect.com/science/article/pii/S1550413113002453) ^3^, Seon-Jin Lee ^5^, Hee Gu Lee ^5^, Won Kon Kim ^3^, In Cheul Jeung ^6^, Nam Woong Song ^2*^, Kwang-Hee Bae ^3*^, and Jeong-Ki Min ^1,4*^

**Affiliations**

^1^ Biotherapeutics Translational Research Center, Korea Research Institute of Bioscience and Biotechnology (KRIBB), 125 Gwahak-ro, Yuseong-gu, Daejeon 34141, Republic of Korea

^2^ Center for Nano-Bio Measurement, Korea Research Institute of Standards and Science (KRISS), 267 Gajeong-ro, Yuseong-gu, Daejeon 34113, Republic of Korea

^3^ Research Center for Metabolic Regulation, KRIBB, 125 Gwahak-ro, Yuseong-gu, Daejeon 34141, Republic of Korea

^4^ Department of Biomolecular Science, KRIBB School of Bioscience, Korea University of Science and Technology (UST), 217 Gajeong-ro, Yuseong-gu, Daejeon 34113, Republic of Korea

^5^ Immunotherapy Convergence Research Center, KRIBB, 125 Gwahak-ro, Yuseong-gu, Daejeon 34141, Republic of Korea

^6^ Department of Obstetrics and Gynecology, College of Medicine, The Catholic University of Korea, 222 Banpo-daero Seocho-gu, Seoul 06591, Republic of Korea

*Corresponding authors contact information: [jekmin@kribb.re.kr](mailto:jekmin@kribb.re.kr) (J.-K. Min), [khbae@kribb.re.kr](mailto:khbae@kribb.re.kr) (K.-H. Bae), nwsong@kriss.re.kr (N.W. Song)

^†^ Contributed equally

**Additional method**

**Preparation of FITC-labeled SiNPs with different sizes**

In order to identify cellular uptake of SiNPs, we fluorescently labeled 20-nm SiNP with fluorescein isothiocyanate (FITC, Sigma Aldrich)- -(3-Aminopropyl)triethoxysilane (APTES, Sigmal Aldrich) conjugate solution. First, FITC-APTES conjugate solution was prepared by mixing 400 µl of butanol (Sigma Aldrich), 2 mg of FITC and 2 drops (using syringe) of APTES. The solution was stirred overnight avoiding light. The mixture of 400 µl of FITC-APTES solution, 12 ml of butanol and 18 ml of TEOS was added to 0.82 µM of L-arginine solution at 50℃. The temperature of the solution was kept at 50 ℃ for 18 h and then only aqueous phase was extracted by the using the separatory funnel.

The FITC-labeled 50-nm SiNP was synthesized in a manner similar to that described above. The FITC-APTES conjugate solution was prepared by mixing 0.12 mg of FITC and 14 mg of APTES in 20 ml of ethanol (Sigma Aldrich). The solution was remained for 24 h under vigorous stirring avoiding light. After completion of the FITC-APTES conjugation, 2.1 ml of Ammonia solution (Sigma Aldrich), 5 ml of FITC-APTES conjugate solution and 0.75 ml of TEOS were added to 44.25 ml of ethanol in the 1 neck round-bottom flask. To wash the FITC-SiNP and change the solvent, centrifugation-wash-sonication process was repeated more than twice and then finally re-dispersed in deionized water.

**Cellular uptake**

The HUVECs were placed on a 60Φ-dish and incubated for 24 hours at 37 °C. After the serum starvation, cells were washed using phosphate-buffered saline (PBS), and media containing 20nm and 50nm SiNPs was added at IC50 value for 12 hours. Then the medium was discarded carefully, the cells were fixed with 3 % formaldehyde and analyzed by a flow cytometry (Beckton Dickenson FACS Caliber, USA).

**Tables**

**Additional table S1.** Size distribution of SiNPs with 20 and 50 nm sizes at 1 mg/ml in various condition containing serum (0.5, 1.0, and 10 %; v/v).

| Concentration of serum (%, v/v) | 20-nm SiNP | |  | 50-nm SiNP | |
| --- | --- | --- | --- | --- | --- |
|  | Size [nm] | Polydispersity (PDI) |  | Size [nm] | Polydispersity (PDI) |
| 0 | 22.4 | 0.088 |  | 51.91 | 0.085 |
| 0.5 | 21.9 | 0.093 |  | 52.03 | 0.096 |
| 1 | 23.8 | 0.123 |  | 97.75 | 0.134 |
| 5 | 44.3 | 0.258 |  | 186.3 | 0.376 |
| 10 | 293.4 | 0.651 |  | 564.8 | 0.473 |

**Figures**

**
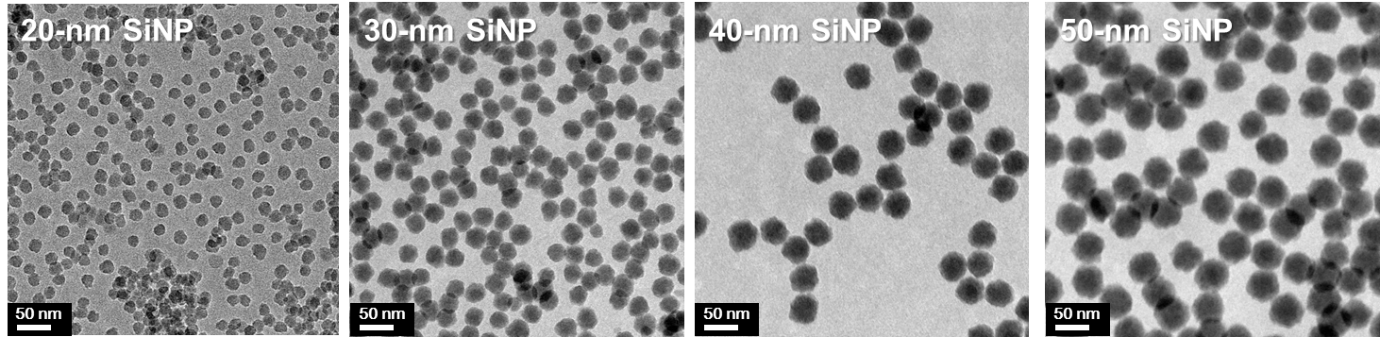
**

**Additional figure S1. Morphology and size distribution of SiNPs.** TEM images of SiNPs with different sizes (scale bar = 50 nm). The images showed that all SiNPs were spherical shape and mono-dispersity. The size of SiNPs were 21.8 ± 0.6^a^, 31.4 ± 2.4, 42.9 ± 2.8, and 56.7 ± 1.5, respectively. ^a)^ Standard deviation was calculated by the uncertainty value.

**
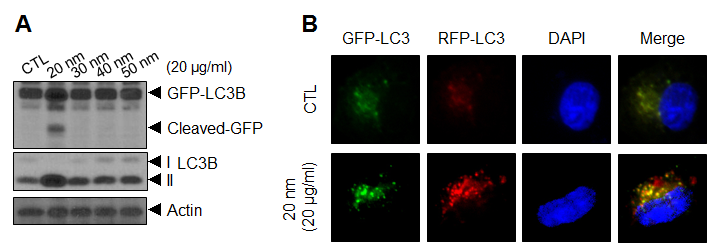
**

**Additional figure S2. GFP fragments were degraded from GFP–LC3 in autolysosomes.** (A) Western blot analysis of cleaved GFP fragments from GFP–LC3. The HUVECs transiently transfected with a GFP–LC3 plasmid were treated with the indicated sizes of 20 μg/ml SiNPs and subjected to Western blotting with an antibody specific for GFP. (B) Representative images of GFP–mRFP–LC3 punctae. Colocalization of GFP and red fluorescent protein (RFP) indicate autophagosomes (yellow dot), whereas only RFP fluorescence indicate autolysosomes (red punctae).


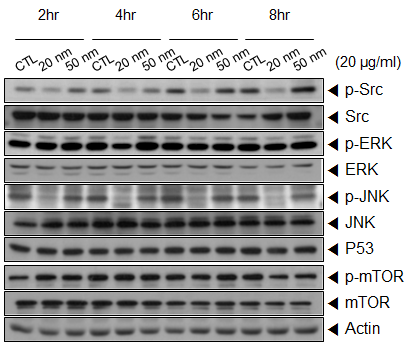


**Additional figure S3. 20nm SiNP-induced autophagy is independent of JNK or p53-mediated AMPK/mTOR signaling pathways.** Western blot analysis of LC3B-I to LC3B-II conversion and phosphorylation of Src, Extracellular signal-regulated kinase (ERK), c-Jun NH(2)-terminal kinase (JNK), p53, and the mammalian target of rapamycin complex 1 (mTOR) in HUVECs treated with the indicated sizes of 20 μg/ml SiNPs in time-dependent manner.


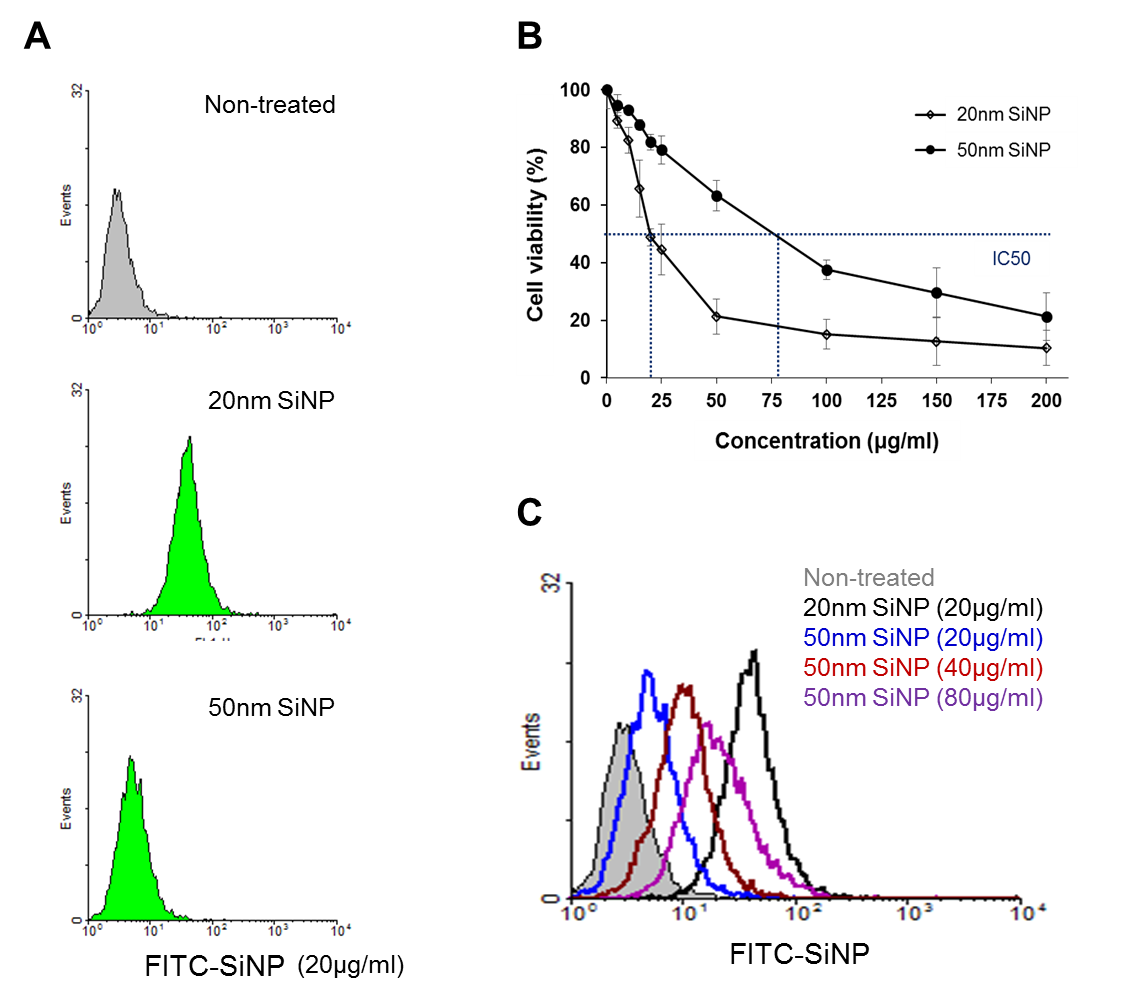


**Additional figure S4. Size and dose-dependent cellular uptake of SiNPs.** (A) Cellular uptake of 20-nm and 50-nm SiNPs with HUVECs was monitored by flow cytometry. (B) Assessment of cell viability following treatment with 20-nm and 50-nm sizes of SiNPs. It was indicated concentrations of the SiNPs for 24 h in low serum-containing condition and analyzed using crystal violet assay. (C) dose-dependent cellular uptake of 50-nm SiNPs with HUVECs was monitored by flow cytometry. Filled histogram and black solid line indicate untreated cells and 20-nm SiNP (20 μg/ml), respectively.


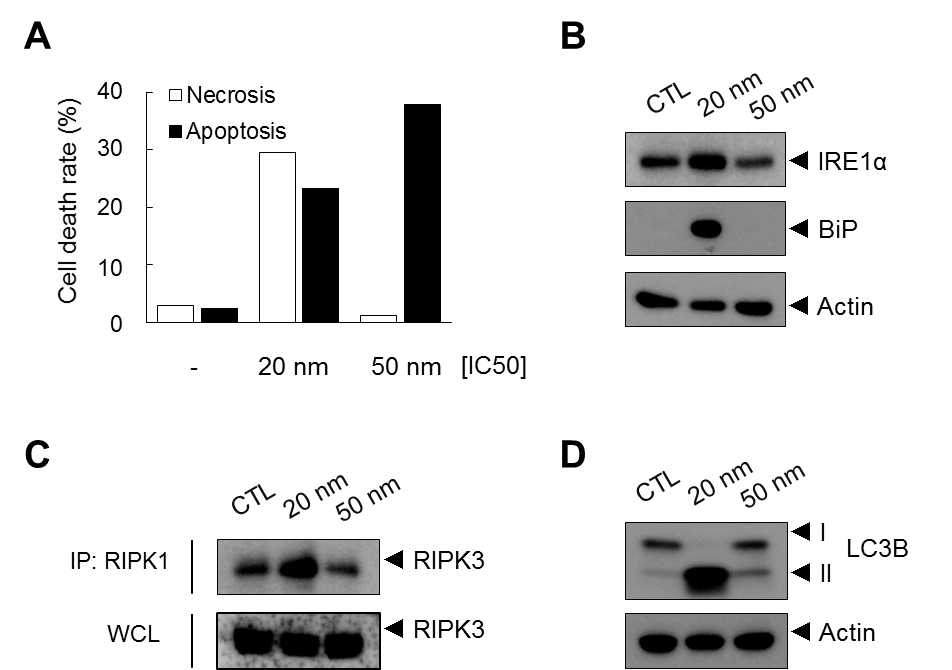


**Additional figure S5. Size-dependent toxic mechanisms of SiNPs.** The HUVECs were treated with SiNPs at the same IC50 value ([20-nm SiNP] = 20 μg/ml & [50-nm SiNP] = 78.2 μg/ml) for 24 h in low serum-containing condition. (A) Representative bar graph of the percentages of apoptotic and necrotic cells as determined by flow cytometric analysis in HUVECs following treatment with SiNPs (early apoptotic cell: annexin-V(+)/PI (­), late apoptotic cell: annexin-V(+)/PI (+), and necrotic cell: annexin-V(­)/PI (+)). (B) Western blot analysis of expression of Binding immunoglobulin Protein (BiP) and inositol-requiring kinase-1α (IRE1α). (C) Interaction between RIPK1–RIPK3 was detected by immunoprecipitation (IP) and western blot analysis. (D) Western blot analysis of LC3-I to LC3-II conversion in HUVECs treated with SiNPs at the IC50 value for 5 h, respectively.
